# Local IGF-1 isoform protects cardiomyocytes from hypertrophic and oxidative stresses via SirT1 activity

**DOI:** 10.18632/aging.100107

**Published:** 2009-12-10

**Authors:** Manlio Vinciguerra, Maria Paola Santini, William C. Claycomb, Andreas G. Ladurner, Nadia Rosenthal

**Affiliations:** ^1^European Molecular Biology Laboratory (EMBL)-Mouse Biology Unit, Campus A. Buzzati-Traverso, Monterotondo-Scalo, Rome 00016, Italy; ^2^ Harefield Heart Science Centre, Imperial College London, Harefield, Middlesex, UB9 6JH, United Kingdom; ^3^ Dept. of Biochemistry and Molecular Biology, Louisiana State Univ. Health Sciences Center, New Orleans, LA 70112, USA; ^4^ European Molecular Biology Laboratory (EMBL)-Genome Biology Unit, Meyerhofstraße, Heidelberg 69117, Germany

**Keywords:** IGF-1, SirT1, oxidative stress, cell hypertrophy, cardiomyocytes

## Abstract

Oxidative and hypertrophic stresses contribute to the pathogenesis of heart failure. Insulin-like growth factor-1 (IGF-1) is a peptide hormone with a complex post-transcriptional regulation, generating distinct isoforms. Locally acting IGF-1 isoform (mIGF-1) helps the heart to recover from toxic injury and from infarct. In the murine heart, moderate overexpression of the NAD^+^-dependent deacetylase SirT1 was reported to mitigate oxidative stress. SirT1 is known to promote lifespan extension and to protect from metabolic challenges. Circulating IGF-1 and SirT1 play antagonizing biological roles and share molecular targets in the heart, in turn affecting cardiomyocyte physiology. However, how different IGF-1 isoforms may impact SirT1 and affect cardiomyocyte function is unknown. Here we show that locally acting mIGF-1 increases SirT1 expression/activity, whereas circulating IGF-1 isoform does not affect it, in cultured HL-1 and neonatal cardiomyocytes. mIGF-1-induced SirT1 activity exerts protection against angiotensin II (Ang II)-triggered hypertrophy and against paraquat (PQ) and Ang II-induced oxidative stress. Conversely, circulating IGF-1 triggered itself oxidative stress and cardiomyocyte hypertrophy. Interestingly, potent cardio-protective genes (adiponectin, UCP-1 and MT-2) were increased specifically in mIGF-1-overexpressing cardiomyocytes, in a SirT1-dependent fashion. Thus, mIGF-1 protects cardiomyocytes from oxidative and hypertrophic stresses via SirT1 activity, and may represent a promising cardiac therapeutic.

## Introduction

In response to age-associated stresses or dysfunction such as pressure/volume overload, myocardial infarction and cardiomyopathies, the heart undergoes adaptation processes that lead to pathological hypertrophy [1]. One of the main causes of cardiac dysfunction and cardiomyocytes loss is an imbalance between the generation of reactive oxygen species (ROS) and the antioxidant defenses in favor of the former [2]. Growing evidence demonstrate that oxidative stress and hypertrophy are mechanistically linked to each other in the heart [3,4]. Several therapeutic strategies are now employed to counteract the deleterious effects of cardiac hypertrophy and oxidative stress, making therefore the analysis of specific cell signaling imperative to generate novel drugs. In this scenario, the insulin like growth factor-1 (IGF-1) and Sirtuin -1 are novel important mediators of cell survival, oxidative stress, regeneration, and life-span regulation [5] in several tissues including the heart.

IGF-1 is a peptide hormone acting as a growth and differentiation factor [6]. The pleiotropic functions of IGF-1 are reflected in the intricate structure of the gene encoding it. The IGF-1 gene spans more than 70 kb, contains two promoters and has six exons, giving rise to multiple splicing variants. These splice variants all consist of the same unvarying core flanked by varying termini. IGF-1 isoforms are classified according to the N-terminal signal peptide (class 1 and 2) and to the C-terminal extension peptides, Ea and Eb [6]. It is established that IGF-1 is both a systemic growth factor produced primarily by liver and a local growth factor functioning in an autocrine/paracrine manner in tissues such as heart and skeletal muscle [6]. Post-transcriptionally, IGF-1 isoforms are cleaved to give a mature 70 amino acid core hormone (identical for all isoforms) devoid of both the signal peptide and the extension peptide. This mature hormone is released into the bloodstream and has been implicated in the restriction of life span [7]. Correspondingly, high levels of circulating IGF1 are associated with increased mortality and cardiovascular diseases in the elderly [8]. When expressed as transgenes in the cardiomyocytes, distinct IGF-1 isoforms result in diverse phenotypes, ranging from protection from hypertrophy to its exacerbation towards pathological states [6,9-11]. The role of IGF-1 in cardiac oxidative stress is also debated: cardiomyocyte-specific IGF-1 overexpression has been reported to protect from angiotensin II (Ang II)-mediated oxidative stress [12] but, on the contrary, severe circulating IGF-1 deficiency, as in hepatocyte-specific IGF-1 knock-out mice, antagonizes oxidative stress and cell death in cardiomyocytes triggered by the potent oxidant agent paraquat (PQ) [13].

The mIGF-1 isoform comprises a Class 1 signal peptide and a C-terminal Ea extension peptide [6]. It is highly expressed in neonatal tissues and in the adult liver, but decreases during aging in extra-hepatic tissues, where its expression is activated transiently in response to local damage [14].  Previous studies from our laboratory showed that continuous expression of mIGF-1 throughout postnatal life did not produce significant perturbations in normal heart physiology and, in contrast to previous studies with other IGF-1 transgenes. [11] did not progress to a pathological phenotype [15]. In response to injury however, molecular analysis revealed that mIGF-1 curtails the inflammatory response, enhances antioxidative cell-defense by upregulation of adiponectin, uncoupling protein 1 (UCP1) and methallothionein 2 (MT-2), and induces cardiac tissue restoration by increasing the number of proliferative cells at the border zone of the infarcted heart [15]. Given the benefits of cardiac restricted mIGF-1 expression, we sought to elucidate the molecular targets of this isoform.

Sirtuin 1 (SirT1) belongs to the sirtuin family of nicotinamide adenine dinucleotide NAD-dependent protein deacetylases, whose activation is considered beneficial for metabolic, neurodegenerative and inflammatory diseases and to augment longevity [5]. Moderate SirT1 activation in murine heart has been shown to protect from oxidative stress and angiotensin II (Ang II)-induced cell death [16,17]. Intriguingly, SirT1 expression is increased in the hypertrophic heart of rodents and monkeys [16,18], although its functional relevance is unclear. IGF-1 and SirT1 share downstream targets in cardiomyocytes, and this in turn may affect cardiovascular function [19]. It has been reported that SirT1 is also activated by the polyphenol resveratrol and by caloric restriction [20], whereas its induction is counteracted by circulating IGF-1 [20]. Moreover, the levels of circulating IGF-1 are lowered upon caloric restriction [21]. Hence, SirT1 and IGF-1 apparently play opposite biological roles, although there is no information on the impact of separate IGF-1 isoforms, acting locally or systemically, on cardiac SirT1. In particular, in this study we sought to test if the liver-produced and fully processed IGF-1 core protein isoform, circulating in the blood stream, and the locally acting mIGF-1 isoform [14], could display distinct effects in the protection from hypertrophic and oxidative stress. We tested this in mouse HL-1 and primary neonatal cardiomyocytes, using Ang II and PQ as hypertrophic and oxidative stressors.

We found that SirT1 and mIGF-1 co-regulate cardiomyocyte survival and protection from damage. mIGF-1 overexpression protects HL-1 cardiac cells and neonatal mouse cardiomyocytes from the deleterious effects induced by hypertrophic (Ang II) and oxidative (PQ) stressors in a SirT1-dependent fashion. The beneficial activity of SirT1 is mediated by the activation of protective molecules such as UCP1, adiponectin and MT2 and is dependent on mIGF-1 expression. Interestingly, the circulating IGF-1 isoform does not regulate SirT1 expression and activity and it is not beneficial during hypertrophy and oxidative stress conditions. The *in vitro* system herein described uncovers a novel signaling cross-talk that suggests potential pharmacological targets to modulate cardiac protection.

## Results

### mIGF-1 increases SirT1 expression and catalytic activity in mouse cardiomyocytes

**Figure 1.**
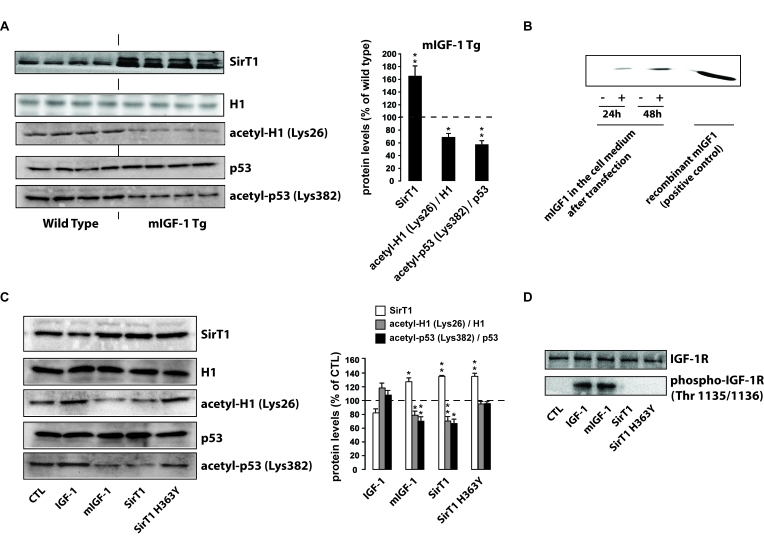
mIGF-1, but not IGF-1, increases SirT1 expression and activity in mouse cardiomyocytes. (**A**) *Left panel*: representative Western blots of SirT1, histone H1, acetyl-H1 (Lys26), detected in nuclear extracts, and of p53 and acetyl-p53 (Lys382), detected in whole tissue lysates, from wild type and mIGF-1 Tg mice. Four animals of a total of 10 are shown; *right panel*: densitometric quantification of SirT1, acetyl-H1(Lys26)/H1 and acetyl-p53(Lys382)/p53 levels in cardiomyocytes from mIGF-1 mice, expressed as % of those in wild type cardiomyocytes. (**B**) representative Western Blot of mIGF-1 detected in the extracellular medium of HL-1 cardiomyocytes, transfected with a plasmid carrying mouse mIGF-1 cDNA. (**C**) *Left panel*: representative Western Blot of SirT1, histone H1, acetyl-H1 (Lys26) detected in nuclear extracts, and of p53 and acetyl-p53 (Lys382) detected in whole cell lysates, from HL-1 cardiomyocytes transfected with the indicated constructs (SirT1 or SirT1 H363Y) or treated with 20 ng/ml IGF-1 for 24 hours; *right panel*: densitometric quantification of SirT1, acetyl-H1(Lys26)/H1 and acetyl-p53(Lys382)/p53 levels in transfected or treated cells, expressed as % of control (CTL). (**D**) Representative Western blots of IGF-1 receptor (IGF-1R) or phospho-IGF-1R (on Thr 1135/1136) in HL-1 cardiomyocytes lysates. Results in (**A**) and (**B**) are means ± SE of 3 independent experiments (^**^^,^^***^p versus unstimulated control cells or untreated WT cardiomyocytes).

It has been reported that SirT1 and IGF-1 share common downstream targets in cardiomyocytes [19], but antagonize each other's activity [20,21] by mechanisms so far unexplored. To elucidate the molecular interplay between the two molecules in cardiac tissue, we examined if SirT1 expression is affected in the heart of mice overexpressing the locally acting mIGF-1 isoform [15]. Analysis of nuclear extracts prepared from whole heart lysates of mIGF-1 transgenic (Tg) and wild type (WT) mice revealed increased SirT1 protein levels in mIGF-1 Tg mouse hearts compared to wild type littermates (Figure 1A). To correlate the overexpression of SirT1 in Tg hearts with its deacetylase activity, we analysed the deacetylation levels of the SirT1 targets, p53 [25] and histone H1 [26]. The  increase in SirT1 expression mediated by mIGF-1 correlated functionally with histone H1  and p53 deacetylation at Lys26 and Lys382 respectively (Figure 1A). To confirm a direct effect of mIGF-1 on SirT1 expression, we overexpressed mouse mIGF-1 in HL-1 cardiomyocytes [22]. mIGF-1 was detected in the cell medium already 24 hours after transient transfection (Figure 1B), and correlated with  increased SirT1 expression (Figure 1C). Interestingly, treatment with 20 ng/ml of the circulating IGF-1, although induced comparable activation of the IGF-1 receptor to that seen with transfected mIGF-1 (Figure 1D), and moderately decreased SirT1 expression (Figure 1C), indicating that mIGF-1 activates differential downstream signaling compared to the circulating peptide. Consistently with the results observed in whole heart lysates from mIGF-1 Tg mice, mIGF-1 overexpression in HL-1 cells promoted decreased deacetylation of H1 and p53 at critical lysine residues (Figure 1C), whereas treatment with circulating IGF-1 induced a moderate upregulation of acetylation levels of both SirT1 targets. Unmodified acetylation levels of p53 and histone H1 were observed in cells overexpressing a catalytic inactive SirT1 protein (H363Y) [20] (Figure 1C). These findings demonstrate that the locally acting mIGF-1 isoform, but not the circulating form, enhances SirT1 expression and activity in cardiomyocytes.

### mIGF-1/SirT1 pathway inhibits Ang II-induced hypertrophic fetal gene expression program

Circulating IGF-1 and SirT1 have both been implicated in the protection against cardiac hypertrophy [10,17], although the role of IGF-1 and/or its isoforms in this process remains controversial [6,9-11]. A molecular hallmark of the progression to cardiac hypertrophy towards heart failure is the re-activation of the ‘fetal' gene program in cardiomyocytes [27]. This process involves an upregulation of genes encoding atrial and brain natriuretic peptides (ANP and BNP), as well as fetal contractile protein isoforms such as α-myosin heavy chain 7 (MYH7) and α-skeletal actin (ACTA-1) is observed. In parallel, cardiac hypertrophy correlates with downregulation of adult α-myosin heavy chain (MYH6) and sarco/endoplasmic reticulum calcium ATPase-2 (SERCA2) [27]. Since Ang II is a potent hypertrophic agonist in cardiomyocyte, inducing re-activation of the fetal gene program [28], we investigated the role of mIGF-1-induced SirT1 expression on the Ang II-dependent fetal gene activation in two different *in vitro* models: in the HL1 cell line, resembling adult cardiomyocytes, and in mouse WT and Tg neonatal cardiomyocytes.

**Figure 2.**
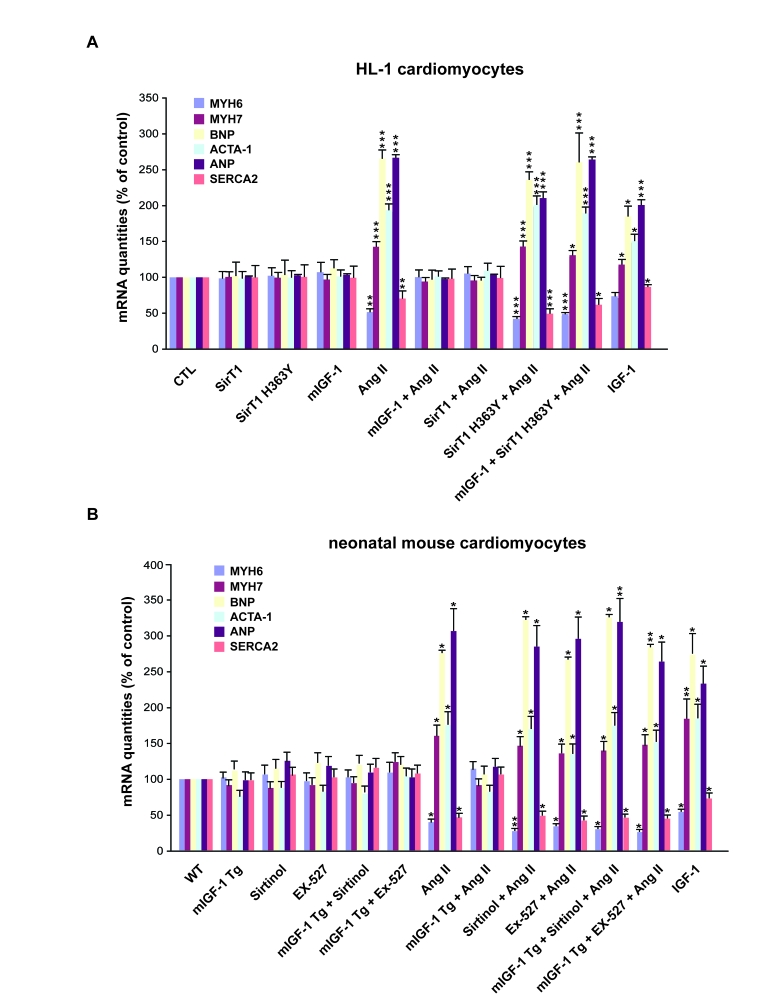
mIGF-1 prevents Ang II- and IGF-1-induced fetal gene program activation. (**A**) HL-1 cardiomyocytes were transfected with the indicated plasmids, or treated with 20 ng/ml IGF-1 for 24 h, before exposure to Ang II (1 μM for 24 h). Untransfected cells were used as control (CTL). (**B**) Neonatal mouse cardiomyocytes from wild type (WT) or heart overexpressing mIGF-1 mice (mIGF-1 Tg) were pre-incubated with sirtinol (100 μM) or EX-527 (1 μM), or treated with 20 ng/ml IGF-1 for 24 h, prior to exposure to Ang II (1 μM for 24 h). Untreated WT cardiomyocytes were used as control. (**A, B**) The expression levels of MYH6, MYH7, BNP, ACTA-1, ANP and SERCA2 mRNAs were examined by qRT-PCR. Results are means ± SE of 3 independent experiments (^*^^,^^**^^,^^***^p versus unstimulated control cells).

When HL-1 cardiomyocytes were exposed for 24 hours to Ang II at 1 μM, a supra-physiological but fairly used concentration to elicit its signaling and hypertrophic effects in cardiomyocytes studies [29,30], the hormone triggered an increase in the mRNA levels of BNP (262±14%), ANP (265±6%), ACTA1 (189±10%) and MYH7 (164±9%) when compared to untreated cells (CTL), and a decrease in SERCA2 (30±9%) and MYH6 (50±4%) transcript levels when compared to CTL cells (Figure 2A). Over-expression of locally acting mIGF-1 or SirT1, but not the catalytic mutant SirT1 H363Y, fully prevented the activation of Ang II-induced fetal gene program (Figure 2A). Importantly, overexpression of mIGF-1 and SirT1 H363Y together did not block the changes in gene expression induced by Ang II, indicating that mIGF-1 protective effect is SirT1-dependent (Figure 2A). In contrast, exposure of HL-1 cells to the circulating form of IGF-1, similarly to Ang II, triggered to some extent the activation of the fetal gene program (Figure 2A).

In a second *in vitro* model, 2 day-old WT and Tg hearts were excised and cardiomyocytes extracted. Cultured cells were exposed for 24 hours to 1 μM Ang II and fetal gene activation analysed by quantitative real-time PCR (qRT-PCR). As expected Ang II treatment increased BNP (276±7%), ANP (306±27%), ACTA (178±15%) and MYH7 (161±16) transcript levels compared to untreated WT cells (Figure 2B). We observed in parallel a decrease in SERCA2 (53±6%) and MYH6 (61±4%) mRNA levels compared to WT untreated cardiomyocytes (Figure 2B). As in the HL-1 cell system,  over-expression of locally  acting mIGF-1 fully prevented the activation of the fetal gene program induced by Ang II (Figure 2B), indicating that mIGF-1 activates antagonist signaling to hypertrophy during the early stages of cardiac development. These data correlates with previous analyses in our laboratory. Although mIGF-1 is known to induce a moderate physiological overgrowth in adult hearts [15], neonatal mIGF-1 expressing hearts do not present increased ANP, BNP and ACTA-1 transcript levels (data not shown). Interestingly, treatment of wild type cardiomyocytes with the circulating IGF-1 induced activation of fetal-like gene expression pattern (Figure 2B).

To examine if the protective effects of mIGF-1 against Ang II-induced fetal gene program in neonatal cardiomyocytes were dependent on SirT1, WT and mIGF-1 Tg cardiomyocytes were treated with two SirT1 pharmacological inhibitors, pan-sirtuin inhibitor (sirtinol, 100 μM) or a SirT1 specific inhibitor (EX-527, 1 μM) [31], prior to exposure to Ang II. At these concentrations, both compounds did not affect cardiomyocyte viability and blocked SirT1 activity, as assessed by increased acetylation levels of its downstream targets p53 (Lys382)  and H1 (Lys26) (data not shown). Upon SirT1 blockade, Ang II treatment induced a significant increase in fetal-like genes in both wild type and mIGF-1 Tg car-diomyocytes, overcoming mIGF-1 protection (Figure 2B).

Taken together, these data support the concept that the locally acting mIGF-1 isoform, but not the circulating liver-produced IGF-1, counteracts the activation of the hypertrophic fetal gene program induced by Ang II in a SirT1-dependent manner.

**Figure 3.**
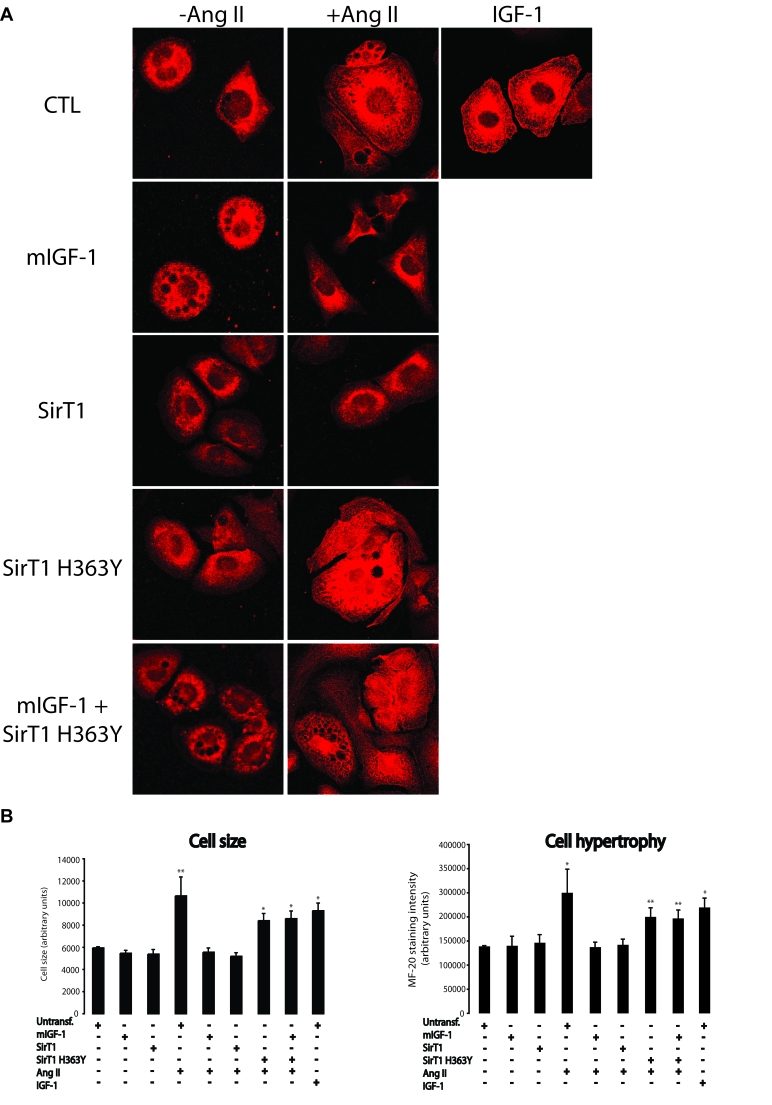
mIGF-1 prevents Ang II- and IGF-1-induced cell hypertrophy (MF-20 staining) in HL-1 cardiomyocytes. (**A**) HL-1 cardiomyocytes were transfected or treated as in Legend of Figure 2A. Sarcomeric myosin was stained with MF-20 antibody and images were acquired using a Leica confocal microscope. (**B**) Cell size and cell hypertrophy quantified according to MF-20 staining in HL-1 cardiomyocytes in the different experimental conditions as in as in Legend of Figure 2A. Results are means ± SE of 3 independent experiments (^*^^,^^**^p versus unstimulated control cells). Bar: 25 μM.

### mIGF-1/SirT1 pathway rescues cell hypertrophy triggered by Ang II or IGF-1

**Figure 4.**
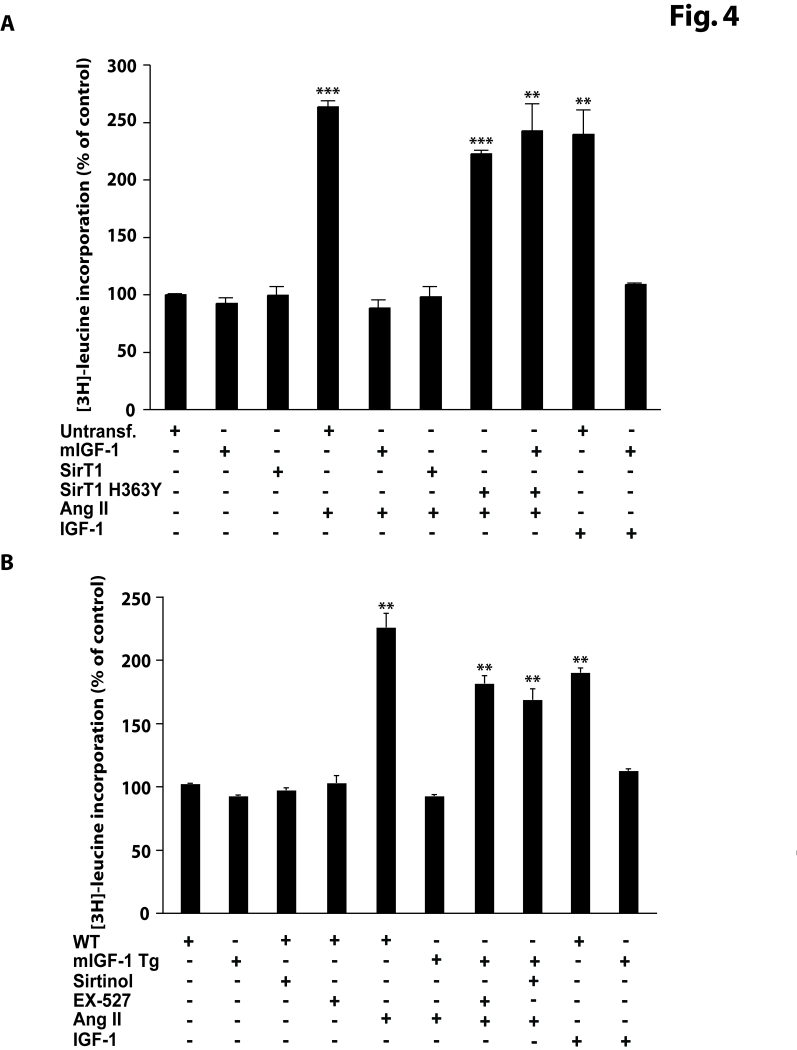
mIGF-1 prevents Ang II- and IGF-1-induced cell hypertrophy ([3H]-leucine incorporation) in HL-1 cardiomyocytes and in mouse neonatal primary cardiomyocytes. (**A**) HL-1 cardiomyocytes were transfected with the indicated plasmids, or treated with 20 ng/ml IGF-1 for 24 h, or exposed to Ang II (1 μM for 24 h). Untransfected cells were used as control (CTL). Together with Ang II, HL-1 cells were also incubated with 1μCi/ml of [3H]-labeled leucine (24 h). (**B**) Neonatal primary cardiomyocytes from wild type or mIGF-1 Tg mice were treated with SirT1 inhibitors (sirtinol, 100 μM; EX-527, 1 μM), or treated with 20 ng/ml IGF-1 for 24 h, or exposed to Ang II  (1 μM for 24 h); concomitantly to Ang II addition, cells were incubated with 1mCi/ml of [3H]-labeled leucine (24 h). (**A, B**) [3H]-leucine incorporation values were normalized to total protein content and expressed as % of control. Results are means ± SE of 3 independent experiments (^**^^,^^***^p versus unstimulated control cells or untreated WT cardiomyocytes).

Cardiomyocyte hypertrophy is typically characterized by cell enlargement and increase in total sarcomeric myosin heavy chain. Here, we sought to determine the impact of mIGF-1-induced SirT1 expression on cell hypertrophy response by two complementary approaches, measure-ment of MF-20 (a monoclonal antibody staining sarcomeric myosin heavy chain) immunoreactivity, and radioactive [3H]-leucine incorporation into cellular proteins. Exposure of HL-1 cardiomyocytes to Ang II led to an increase in cell size as assessed by MF-20 staining intensity (Figure 3A and B). Surprisingly,  about 30%  of total HL-1 cells died when treated with this hormone (see Figure 8), indicating that Ang II is both a pro-apoptotic and pro-hypertrophic agonist at 1 μM concentration. When mIGF-1 or SirT1 were overexpressed in HL-1 cells, a full blockade of cell size increase induced by Ang II was observed (Figure 3A and B), whereas the catalytic inactive SirT1 H363Y was unable to prevent Ang II-triggered cell hypertrophy (Figure 3A and 3B).  Interestingly, the circulating IGF-1 isoform led to HL-1 cell hypertrophy (Figure 3A and 3B). Similar results were obtained with [3H]-leucine incorporation experiments (Figure 4A), confirming that mIGF-1 induced SirT1 activity prevents Ang II- and IGF-1-induced cell hypertrophy in HL-1 cardiomyocytes.

**Figure 5.**
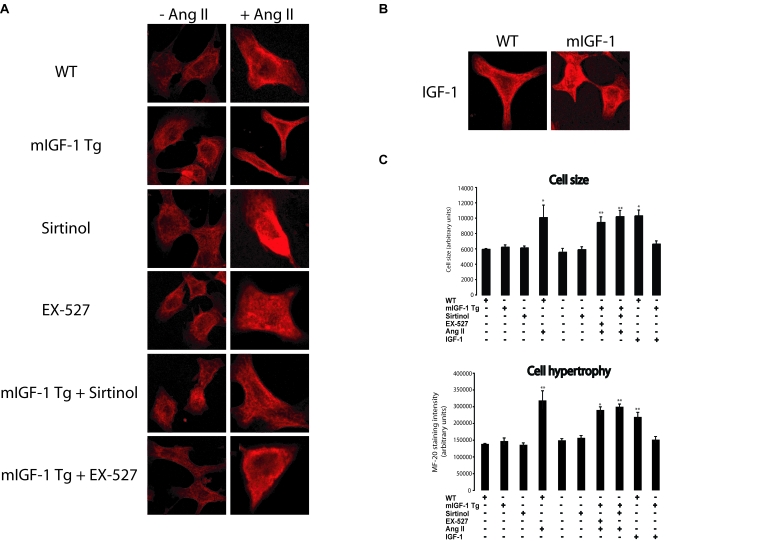
mIGF-1 prevents Ang II- and IGF-1-induced cell hypertrophy (MF-20 staining) in mouse neonatal primary cardiomyocytes. (**A**) Neonatal primary cardiomyocytes from wild type or mIGF-1 Tg mice were treated as in Legend of Figure 2B. (**B**) Cell size and hypertrophy were quantified according to MF-20 staining in the different experimental conditions as in as in Legend of Figure 2B. Results are means ± SE of 3 independent experiments (^*^^,^^**^p versus unstimulated control cells). Bar: 25 μM.

The effect of SirT1 in mIGF-1-dependent protection from cell hypertrophy was investigated as well in neonatal primary cardiomyocytes (Figure 5A-C and Figure 4B). mIGF-1 Tg cardiomyocytes were unresponsive to Ang II-induced cell hypertrophy as indicated by MF-20 staining (Figure 5A and 5B), while blocking SirT1 activity with sirtinol or EX-527 restored Ang II-induced hypertrophy (Figure 5A and 5B), indicating that mIGF-1 inhibitory effect on Ang II- induced cell hypertrophy is dependent on -SirT1 activity also in primary cardiomyocytes. In addition, exposure of wild type neonatal cardiomyocytes to the circulating form of IGF-1, triggered cell hypertrophy (Figure 5A and 5B). Similar findings were observed with [3H]-leucine incorporation experiments (Figure 4B). Therefore, using two different experimental approaches, we found that SirT1 activity induced by mIGF-1, but not by liver-produced IGF-1 isoform, displays antihypertrophic effects in mouse cardiomyocytes.

**Figure 6.**
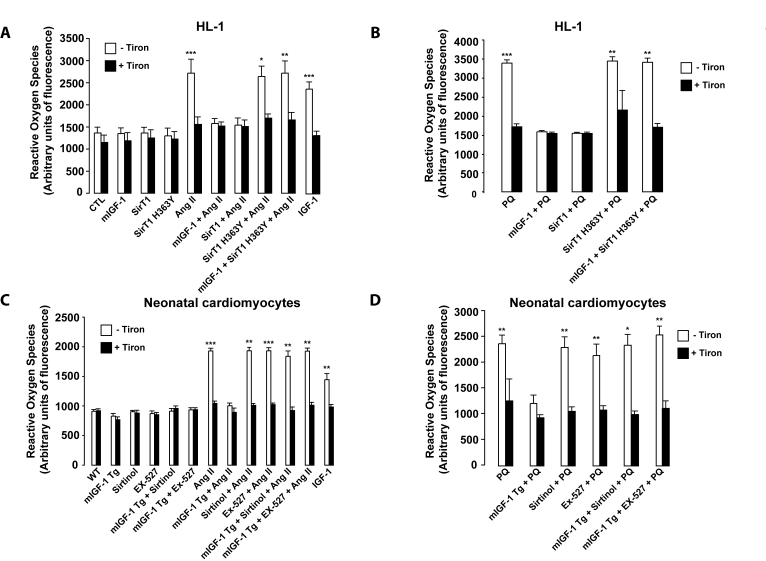
mIGF-1 prevents Ang II-, PQ- and IGF-1-induced increase in reactive oxygen species (ROS) generation in HL-1 cardiomyocytes and in mouse neonatal primary cardiomyocytes. (**A, B**) HL-1 cardiomyocytes were transfected or treated as in Legend of Figure 2A, except that Ang II (1 μM) or PQ (100 μM) were added for only 60 min. Untransfected cells were used as control (CTL). (C, D) Neonatal primary cardiomyocytes from wild type or mIGF-1 Tg mice were treated as in Legend of Figure 2A, except that Ang II (1 μM) or PQ (100 μM) were added for only 60 min. (**A-D**) ROS production was monitored with the fluorescent probe dichlorofluorescein diacetate (CM-DCFDA) and fluorescence values were normalized to protein content. Results are means ± SE of 3 independent experiments (^*^^,^^**^^,^^***^p versus unstimulated control cells or untreated WT cardiomyocytes).

### mIGF-1/SirT1 pathway prevents reactive oxygen species (ROS) generation, peroxidation products and cell death triggered by oxidative stressors

**Figure 7.**
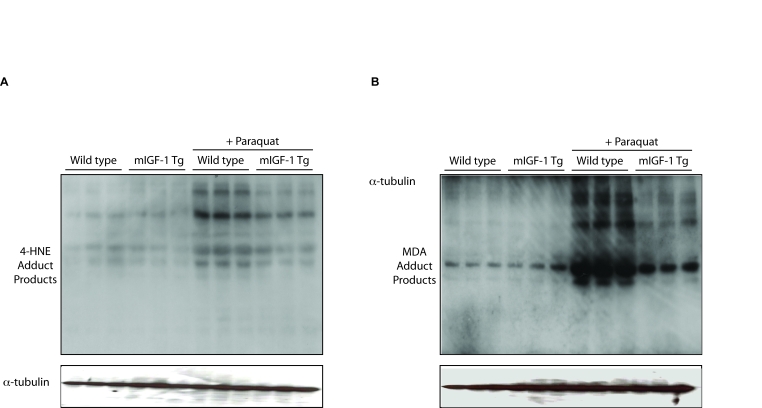
mIGF-1 protects the murine heart from PQ-induced oxidative stress. PQ was injected intraperitoneally at a concentration of 30 mg/kg, while control animals were injected with a saline solution. All mice were sacrificed 24 hours after injections. The figure shows representative Western blots of 4-hydroxy-2-nonenal (4-HNE) adduct products (*left panel*) and of malondialdehyde (MDA) adduct products (*right panel*) from wild type, mIGF-1 Tg, wild type plus PQ and mIGF-1 mice plus PQ. Three animals of a total of 10 are shown in both panels **A** and **B**.

ROS generation and oxidative stress contribute to the progression of pathological cardiac hypertrophy and heart failure. Indeed, oxidative stress and hypertrophy are intimately linked in cardiac muscle[3]. It is increasingly appreciated that the Ang II hypertrophic effects on cardiomyocytes are strictly dependent on the generation of ROS [32]. IGF-1 also triggers ROS production, although it is controversial if this growth factor antagonizes or favors oxidative stress in cardiomyocytes[12,13]. Since SirT1 overexpression has been reported to protect the murine heart from PQ-induced oxidative stress [16], we measured ROS content by dichlorofluorescein diacetate (CM-DCFDA) method in mouse cardiomyocytes to shed light on the impact of IGF-1/SirT1 signaling on oxidative stress generated by Ang II and by PQ. To this end, HL-1 or neonatal cardiomyocytes were pretreated with superoxide anion scavenger Tiron before exposure to Ang II or PQ for 1 hour (Figure 6A-D). In both cardiomyocytes models, Ang II and PQ triggered a significant augmentation in intracellular ROS compared to untreated control cells, which was fully blocked by Tiron (Figure 6A-B, and 6C-D, for HL-1 and neonatal cardiomyocytes, respectively). mIGF-1 did not induce ROS production and efficiently prevented ROS generation by Ang II and PQ in both cardiomyocytes models (Figure 6A-D). Similarly, also SirT1 overexpression reversed ROS production in HL-1 cardiomyocytes (Figure 6A and B). In addition, blocking SirT1 enzymatic activity, by overexpression of SirT1 H363Y in HL-1 cells or incubation with SirT1 inhibitors in neonatal cardiomyocytes, abrogated the protective effects of mIGF-1 against Ang II- and PQ-induced intracellular ROS generation (Figure 6A-D). In contrast to locally acting mIGF-1 isoform, incubation of cardiomyocytes with the circulating IGF-1 isoform triggered a significant rise in ROS content, however less sustained than that generated by Ang II or by PQ (Figure 6A and C). Taken together, these data clearly indicate that mIGF-1, but not IGF-1, shields mouse cardiomyocytes from a rise of intracellular ROS generated by oxidative stressors.  To ascertain if mIGF-1 exerts a cardio-protective role against oxidative stress as well *in vivo*, we injected peritoneally wild type and mIGF-1 Tg mice with PQ, and we assessed lipid and protein peroxidation levels, normally increasing upon ROS generation in cardiomyocytes [33]. Immunoblot analyses of lipid peroxidation 4-hydroxy-2-nonenal (4-HNE) and malondialdehyde (MDA) protein adducts in the heart showed that the levels of both protein adducts were significantly increased in the heart of PQ-injected wild type mice, whereas hearts of mIGF-1 Tg mice were to some extent protected from forming these compounds upon PQ injection (Figure 7). These data indicate that mIGF-1 protects the murine heart from oxidative stress as well *in vivo*.

**Figure 8.**
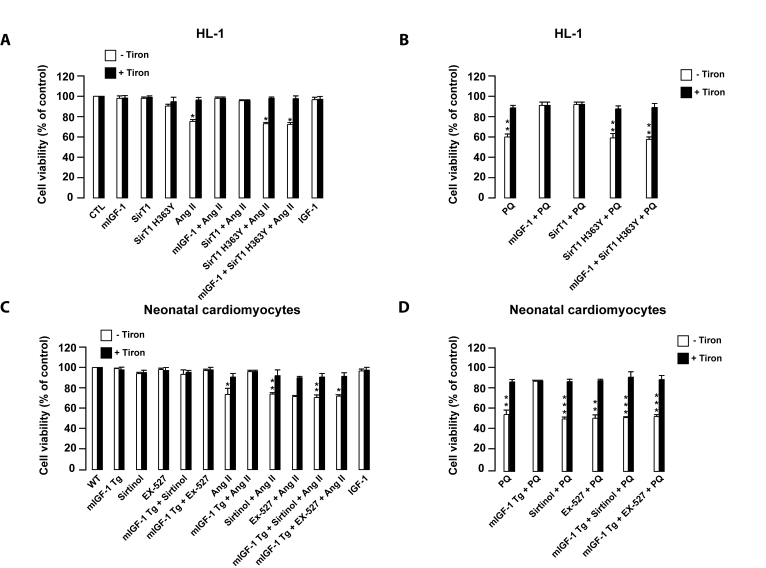
mIGF-1 prevents Ang II- and PQ-dependent cell death in HL-1 cardiomyocytes and in mouse neonatal primary cardiomyocytes. (**A, B**) HL-1 cardiomyocytes were transfected or treated as in Legend of Figure 2A. (**C, D**) Neonatal primary cardiomyocytes from wild type or mIGF-1 Tg mice were treated as in Legend of Figure 2B. (**A-D**) Cell viability was monitored with propidium iodide (PI) by flow cytometry and values were normalized to protein content. Results are means ± SE of 3 independent experiments (^*^^,^^**^^,^^***^p versus unstimulated control cells or untreated wild type cardiomyocytes).

ROS-mediated oxidative stress may lead to cardiomyocyte cell death [34]. Therefore, we examined if ROS production induced by Ang II, PQ and IGF-1 contributed to mouse cardiomyocyte cell necrosis and examined the role of mIGF-1/SirT1 signaling in this process. HL-1 or neonatal mouse cardiomyocytes were preincubated with Tiron before adding Ang II or PQ for 24 hours (Figure 8A-D). Consistently with ROS data, Ang II and PQ induced necrosis in 30% and 50% of the total cell population respectively, as assessed by flow cytometry with propidium iodide (PI) (Figure 8A-B, and 8C-D, for HL-1 and neonatal cardiomyocytes, respectively). mIGF-1 had no effect on cardiomyocyte viability and efficiently prevented Ang II- and PQ-induced necrosis (Figure 8A-D). Moreover, SirT1 overexpression protected HL-1 cardiomyocytes from Ang II- and PQ-dependent cell necrosis (Figure 8A and B). When SirT1 activity was inhibited by sirtinol or by SirT1 H363Y in both HL-1 cells and neonatal cardiomyocytes, no beneficial effect of mIGF-1 to Ang II- and PQ-induced cell death was observed (Figure 8 A-D). Interestingly, despite generating intracellular ROS, the circulating IGF-1 isoform did not impact cell viability (Figure 8A and C). In summary, mIGF-1/SirT1 signaling protects cardiomyocytes from cell death caused by sustained exposure to oxidative stressors.

**Figure 9.**
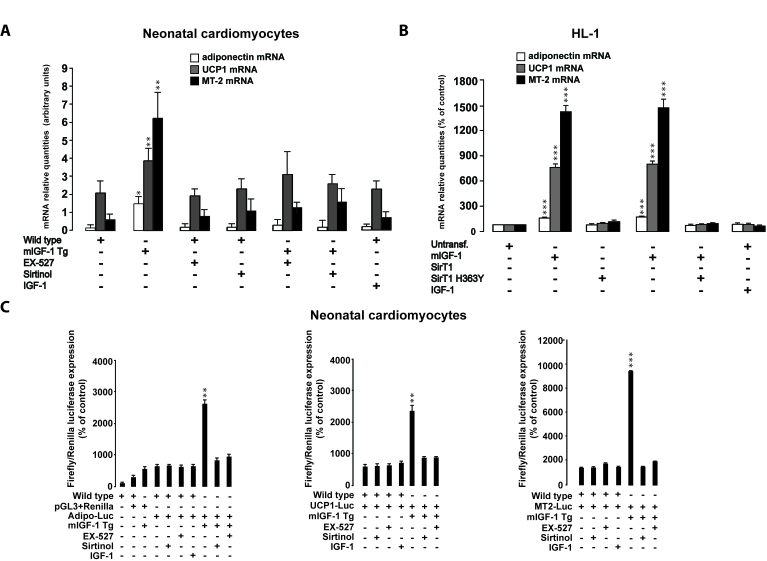
SirT1 is necessary for mIGF-1-dependent upregulation of anti-oxidant and hypertrophic genes adiponectin, UCP1 and MT-2. (**A**) Neonatal primary cardiomyocytes from wild type or mIGF-1 Tg mice were treated with sirtinol (100 μM) or EX-527 (1 μM), or treated with 20 ng/ml IGF-1 for 24 h. (**B**) HL-1 cardiomyocytes were transfected with the indicated plasmids, or treated with 20 ng/ml IGF-1 for 24 h. Untransfected cells were used as control (CTL). (**A, B**) The expression levels of adiponectin, UCP-1 and MT-2 mRNAs were examined by Real Time-PCR. (**C**) Neonatal primary cardiomyocytes from wild type or mIGF-1 Tg mice, and HL-1 cardiomyocytes, were transfected with 1 μg of plasmids carrying Firefly luciferase under the control of promoters of adiponectin (Adipo-Luc), UCP1 (UCP1-Luc) and MT-2 (MT-2-Luc) genes, respectively, together with 1 μg of Renilla Luciferase plasmid. Neonatal primary cardiomyocytes were also treated with different inhibitors or IGF-1 as described in (**A**). Dual luciferase assays were performed in duplicate for each condition.  (**A-C**) Results are means ± SE of 3 independent experiments (^*^^,^^**^^,^^***^p versus untreated cardiomyocytes).

### Activation of cardio-protective genes by mIGF-1/SirT1

Next, we examined if the activation of cardio-protective mediators/effectors by mIGF-1 is dependent on SirT1 signaling, mining our previous Affymetrix analysis of mRNA transcripts in the heart of mIGF-1 Tg mice versus wild type littermates [15]. Among the upregulated transcripts in the heart of mIGF-1 Tg mice, we focused on three cardio-protective genes whose expression was significantly (2- to 4- fold) increased: adiponectin, UCP-1 and MT-2 [35-37]. Increased cardiac expression of UCP1, MT2 and adiponectin mRNA levels was confirmed in mIGF-1 transgenic hearts compared to WT (Figure 9A). In cardiomyocytes from mIGF-1 Tg mice, inhibition of SirT1 activity lowered adiponectin, UCP-1 and MT-2 mRNAs to wild type levels, indicating that their upregulation by mIGF-1 is tightly dependent on SirT1 activity (Figure 9A). On the other hand, exposure of neonatal cardiomyocytes to circulating IGF-1 did not alter adiponectin, UCP-1 and MT-2 mRNA levels (Figure 9A). Consistently, overexpression of mIGF-1 in HL-1 cardiomyocytes led to significantly increased mRNA levels of adiponectin, UCP-1 and MT-2, while IGF-1 had no effect (Figure 9B). SirT1 overexpression in HL-1 cardiomyocytes also triggered an increase in these mRNAs (Figure 9B). Conversely, overexpression of SirT1 H363Y did not affect mRNA expression of these genes and blocked their upregulation by mIGF-1 (Figure 9B).

**Figure 10.**
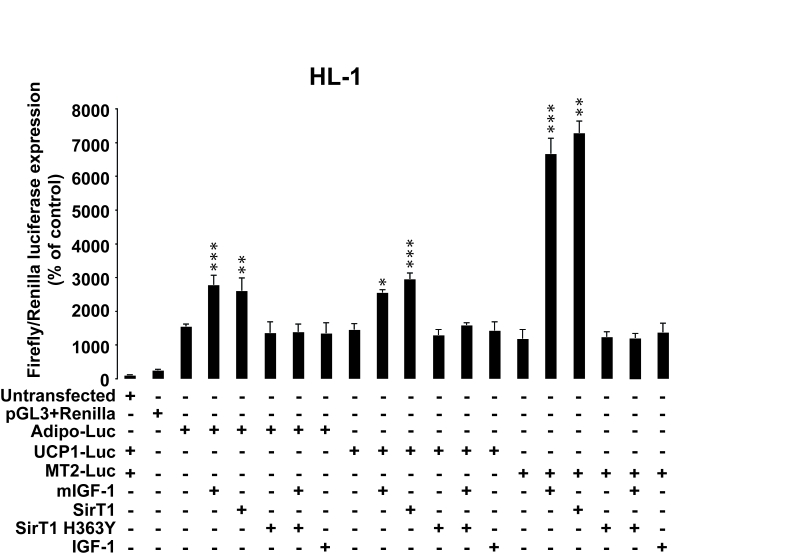
SirT1 is necessary for promoter-dependent mIGF-1-dependent upregulation of anti-oxidant and hypertrophic genes adiponectin, UCP1 and MT-2. HL-1 cardiomyocytes were transfected with the indicated plasmids, and/or treated with 20 ng/ml IGF-1 for 24 h. HL-1 cardiomyocytes were also co-transfected with 1 μg of plasmids carrying Firefly luciferase under the control of promoters of adiponectin (Adipo-Luc), UCP1 (UCP1-Luc) and MT-2 (MT-2-Luc) genes, respectively, together with 1 μg of Renilla Luciferase plasmid. Untransfected cells were used as control. Dual luciferase assays were performed in duplicate for each condition. Results are means ± SE of 3 independent experiments (^*^^,^^**^^,^^***^p versus untransfected/unstimulated control cells).

To elucidate if mIGF-1 could upregulate mRNA levels through SirT1-dependent promoter activation, we transiently transfected neonatal cardiomyocytes and HL1 cells with constructs carrying the minimal promoter region of the three genes, driving the firefly luciferase expression (see Table 2). The analysis showed that mIGF-1/SirT1  pathway activates the expression of  these genes, as indicated by a substantial increase in luciferase activity (Figure 9C and Figure 10). Luciferase activation by mIGF-1 was tightly dependent on SirT1 function, since inhibition strategies (overexpression of SirT1 H363Y in HL-1 cardiomyocytes and SirT1 inhibitors in neonatal cardiomyocytes) blocked the increase in adiponectin, UCP-1 and MT-2- promoter-driven luciferase activity (Figure 9C and Figure 10). In contrast to mIGF-1 isoform, the circulating form of IGF-1 did not alter the promoter activity of adiponectin, UCP-1 and MT-2 in mouse cardiomyocytes (Figure 9C and Figure 10). We conclude that mIGF-1, but not IGF-1, activates at least some cardio-protective genes through SirT1-dependent activation of their promoters.

**Figure 11.**
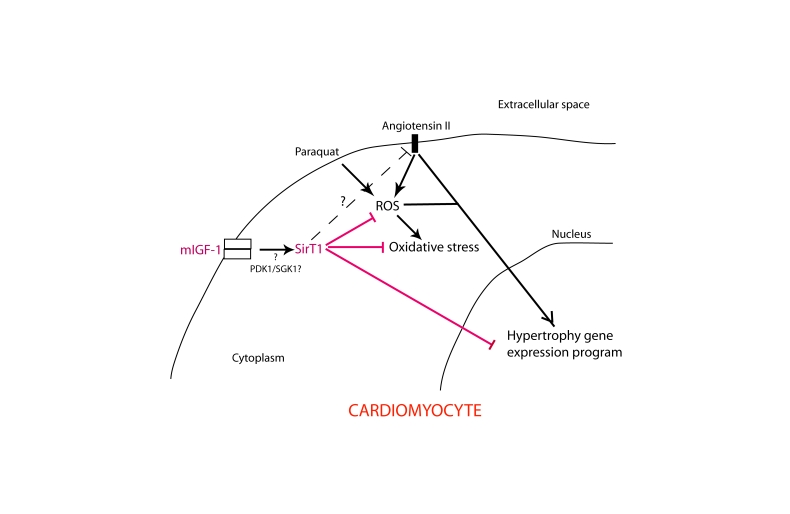
Simplified scheme illustrating the role of mIGF-1-induced SirT1 activity in protection against Ang II- and PQ-mediated oxidative stress and hypertrophy in cardiomyocytes. Question point and dashed line indicate unanswered issues and hypothetical signaling  cross-talk, respectively.

## Discussion

Hypertrophy and oxidative stress are intertwined processes in cardiomyocytes [3,4], contributing to heart disease progression [1,2]. In this study, we have identified a signaling pathway efficiently protecting mouse cardiomyocytes from oxidative and hypertrophic stresses (Ang II and PQ) that relies on the activation of NAD-dependent deacetylase SirT1 by the locally acting mGF-1 isoform. We show that mIGF-1-dependent SirT1 activation reduces ROS levels and cell death triggered by Ang II and PQ, and prevents Ang II-induced hypertrophic response (Figure 11). Our report is consistent with others showing that SirT1 display cardio-protective effects against oxidative stress-dependent cell death [16-18], and that SirT1 may elicit protection from cell hypertrophy by restoring MYH7 expression [38]. Interestingly, in smooth muscle cells SirT1 inhibits the expression of Ang II type 1 receptor [39], but if a similar mechanism occurs in cardiomyocytes remains to be established (Figure 11). We demonstrated that SirT1 counteracts both Ang II-induced cardiomyocyte hypertrophy and ROS-dependent cell death. The dual roles of Ang II as a prohypertrophic and pro-apoptotic agent may rely on the cross-talk between the EGF receptor and the different PI3K isoforms (a, b, gand δ) [40], leading in turn to hypertrophic growth or alternatively to cell death.Recently, it has been shown that other sirtuins family members (SirT3 and SirT7), play an important protective role against cardiac pathology [41,42], indicating that mIGF-1-mediated protective effects against oxidative and hypertrophic stresses could be in part due to other members of this family. However, our data showed that a specific SirT1 inhibitor (EX-527) or overexpression of dominant negative SirT1 protein (H363Y) can reverse mIGF-1 protective effects, supporting a SirT1-specific mechanism.

Importantly, our analysis showed that the locally produced mIGF-1 and the circulating IGF-1 have different roles in SirT1-mediated activity and cardiac protection. Although circulating IGF-1 and mIGF-1 trigger phosphorylation of the same receptor(s) (Figure 1C), differences in their respective signaling mechanisms leading to changes in SirT1 expression/activity must rely downstream of IGF-1 receptor(s). In this respect, it is important to stress that while circulating IGF-1 activates typically PI3K/AKT/mTOR and MAPK pathways [43], locally acting mIGF-1 does not activate these canonical pathways in cardiomyocytes, impinging instead on PDK1 and SGK1 signaling [15]. Thus, divergent signaling mechanisms could explain the apparently antagonistic roles of the two IGF-1 isoforms in cardiomyocytes, with mIGF-1 able to prevent circulating IGF-1-induced cell hypertrophy. We have previously reported that mIGF-1 induced accelerated cardiac growth, related to higher expression levels of ANP at 1 and 2 months, without any further significant change [15]. Interestingly, in the *in vitro* models herein described mIGF-1 did not elicit increased hypertrophic markers and cell size in both neonatal and adult (HL1) cardiomyocytes, indicating that the *in vivo* response is mainly due to specific physiological signaling occurring during cardiac development. Further work using *in vivo* and *in vitro* cardiac models is necessary to shed light on the intermediate players between mIGF-1-dependent signaling and SirT1 in cardiomyocytes (Figure 11). It would be important to understand whether some specific effects of mIGF-1 could be recapitulated by its N-terminal Class 1 signaling or C-terminal Ea extension peptides alone [6], which are absent in cleaved circulating IGF-1. It would be of interest also to ascertain if our data on mIGF-1/SirT1-dependent protection against oxidative and hypertrophic challenges can be confirmed in an *in vivo* setting, where circulating and autocrine/paracrine factors, absent in cultured cell systems, may have an impact.

mIGF-1 Tg mice display activation in the heart of genes involved in anti-apoptotic and anti-oxidant defenses [15]: among the most upregulated, we focused on adiponectin, UCP-1 and MT-2 [15]. Although these proteins are functionally unrelated (adiponectin is a hormone regulating metabolic processes, UCP-1 is a mitochondrial protein allowing protons to reenter the mitochondrial matrix short-circuiting the respiratory chain, and MT-2 is a zinc-binding protein), remarkably they have been reported independently to exert protection against hypertrophic and oxidative stresses in the heart [35-37]. Strikingly, we found that the activation of these genes by mIGF-1 relies on SirT1-dependent activation of their promoters, suggesting that at least some of the mIGF-1 dependent transcriptional program in cardiomyocytes is mediated by SirT1. Our data are in agreement with the finding that SirT1 upregulates adiponectin [44], whereas to our knowledge this is the first report about the SirT1-dependent regulation of UCP-1 and MT-2 transcripts.

In conclusion, there is increasing evidence that NAD-regulated enzymes such as SirT1 finely interplay in the regulation of cardiomyocyte function [17,45], and their role begins now to be appreciated. Consequently, research on the role of IGF-1 isoforms in this "NAD world" is also in its infancy. This domain is considered of clinical interest for the treatment of cardiovascular diseases [5,46], and the mIGF-1/SirT1 pathway presented in this study may represent a promising therapeutic target to fight cardiac hypertrophy and oxidative stress.

## Materials and methods

Animals. Transgenic FVB mice carrying a rat mIGF-1 cDNA driven by the mouse α-MyHC promoter were generated and maintained as previously described [11].

Western analyses. Protein extraction from whole cell or heart tissue preparation was performed in RIPA buffer (1% (w/w) Nonidet P40, 1% (w/w) Sodiumdeoxycate, 0.1% (w/v) SDS, 150mM NaCl, 50mM HEPES pH 7.0, 2mM EDTA pH 8.0, 100mM NaF, 10% glycerol, 1.5mM MgCl2, 100mM PMSF in ETOH, 200mM sodium orthovanadate, 1 μg/ml aprotinin). For analyses of nuclear proteins (SirT1, H1), nuclear fraction was isolated from cultured cells or heart tissues according to the following procedure:  cells or liquid nitrogen-powderized heart tissues were dissolved in buffer A (10 mM HEPES, 1.5 mM MgCl2, 10 mM KCl, 0.5 mM DTT, 0.05% NP-40, 100mM PMSF in ETOH, 200mM sodium orthovanadate, 1 μg/ml aprotinin, pH 7.9) and left on ice for 10 min. After centrifugation, cytoplasmic fraction (supernatant) was kept aside and frozen. Pellets were resuspended in buffer B (5 mM HEPES, 1.5 mM MgCl2, 0.2 mM EDTA, 0.5 mM DTT, 26% glycerol (v/v), 100mM PMSF in ETOH, 200mM sodium orthovanadate, 1 mg/ml aprotinin, pH 7.9) plus NaCl to give a final concentration of 300mM NaCl. Lysates were then mechanically homogenized with a Dounce homogenizer on ice; samples were left on ice for 30 min. After a final centrifugation, supernatant (nuclear fraction) was collected for further analysis. Protein concentration was determined using Bradford method (Biorad) and 20 mg of protein lysates were separated in SDS polyacrylamide mini-gel (Biorad system) and transferred onto a hybond ECL nitrocellulose membrane (Amersham). Membranes were blocked with 5% milk, blotted with specific antibodies o/n at 4oC, washed 3 times with washing buffer (TBS and 0,1% Tween-20) for 30 min and blotted with specific secondary antibodies (horseradish peroxidase-conjugated, 1:5000) with 5% milk for 1h at RT. The membrane was incubated for 1 min using ECL reagent before exposure.

Cell cultures, transfections. Cardiac muscle cell line HL-1 was cultured as previously described, on gelatin/fibronectin coated flasks or multi-wells plates [22]. For transient plasmid transfection or co-transfection experiments, the lipid-based reagent LipofectamineTM 2000 (Invitrogen) was used, according to manufacturer instructions. For luciferase assays, 2 x 106 cells/well were transfected with 1 μg of luciferase reporter constructs and 1μg of pRL-TK (Renilla luciferase construct from Promega). Luciferase assays were performed 48 hours after transfection using a dual-luciferase reporter assay (Promega) and a luminescence counter VictorTM Light 1420 (Perkin Elmer). Firefly luciferase activity was normalized to renilla luciferase expression for each sample.

Preparation of primary neonatal cardiomyocytes culture. One-day-old C57/Bl6 or mIGF-1 transgenic mice were sacrificed and hearts were excised. After scalpel homogenization, ventricular cardiomyocytes were isolated following a series of collagenase/pancreatin digestions (Collagenase type II, CSL2, Worthington/Pancreatin 4x NF, GIBCO) and cells were collected by centrifugation (8.000rpm for 5min). Next, fibroblasts were removed from the culture after a 45 min pre-plating step at 37°C in complete medium [DMEM/199 medium (5/1 ratio) supplemented with 10% heat inactivated horse serum (Sigma), 5% heat inactivated fetal calf serum (Sigma), 0.025 M HEPES, 0.002M L-glutamine (Sigma) and 1x penicillin/streptomycin (Sigma)]. Alive cardiomyocytes were counted using Tryptan Blue solution (Sigma). Cells were transferred on 1% gelatin (Sigma) -coated 12- or 96-well plates.

Reactive oxygen species (ROS) measurements. The fluorescent probe dichlorofluorescein diacetate (CM-DCFDA, Sigma) was used to monitor the intracellular generation of reactive oxygen species (ROS). HL-1 or neonatal mouse cardiomyocytes, grown on coated 96-wells plates were transfected and/or treated for 60 minutes with Angiotensin II (1 mM, 60 min) or paraquat (100 mM) as described, with or without 10 mM superoxide scavenger Tiron. After washing with PBS, cells were incubated 20 min in the dark with 10 mM CM-DCFDA. Cells were washed again with PBS and fluorescence was detected at excitation/emission wavelength of 485-535nm in a fluorimeter Fluoroskan Ascent PL (Labsystems). Fluorescence values were normalized to protein content for each well.

[3H]-leucine incorporation. The cells (HL-1 or neonatal mouse cardiomyocytes) were plated on 12-well-coated dishes at a density of 100 cells/mm2. Protein synthesis was measured by [3H] Leucine (1 μCi/ml) incorporation as described elsewhere [23].

MF-20 immunostaining and confocal microscopy. Cells (HL-1 or neonatal mouse cardiomyocytes) were plated on coated coverslips. Upon the indicated treatment/transfection, cells were washed twice in PBS and fixed with 4% paraphormaldheyde for 10 min ice. Blocking was performed in PBS calcium free plus 10% goat serum, followed by 1 h incubation at RT with the MF-20 antibody diluted 1/250 in PBS calcium free plus 1.5% goat serum, and by 45 min incubation at RT with secondary Cy3 antibody (red) diluted 1/300 in PBS plus 1%BSA and 0.2% Triton-X. Coverslips were mounted on microscopy slides and confocal images were acquired on a Leica TCS SP5 microscope. Cell size (total area) and cell hypertrophy (total MF-20 staining intensity) were accurately quantified using the Metamorph® imaging software (Molecular Devices).

Cell viability assay. Cell viability/cell death was quantified by staining HL-1 cells or neonatal mouse cardiomyocytes with propidium iodide (Invitrogen) following cell transfections and/or treatments as indicated. Fluorescent intensity was analyzed using the BD FACSCanTM System. All FACS data was analyzed with FlowJo (Tree Star, USA).

Real-Time PCR. Total RNA was isolated from hearts using TRIzol (Invitrogen). Afterwards, the RNA was treated with DNaseI enzyme (Promega) for 1h at 37oC and then cleaned by column purification (Qiagen). The RNA concentration was determined with a spectrophotometer. After RNA quality verification, 1-2 mg was used to prepare cDNA (Ready-To-Go, T-Primed First-Strand Kit, Amersham Bioscience). Quantitative polymerase chain reaction (PCR) was performed using the SYBR Green (SIGMA) in a Light-Cycler (Roche). UbiC, Rn18S and GAPDH transcripts were used as internal controls, according to the GeNorm method [24]. Primer sequences were designed with the Primer 3 software (http://frodo.wi.mit.edu/) and are listed in Table 1.

Statistical analysis. Results are expressed as means ± S.E. Comparisons were made by using appropriated Student's t test. Differences were considered as significant when P<0.05 (*), P<0.01 (**) or P<0.001 (***).

Reagents, antibodies and plasmids. All reagents, antibodies and plasmids not described elsewhere in the text are listed below in Table 2.

**Table 1.** Primers sequences for real-time PCR.

**Mouse**	**Forward**	**Reverse**
**SirT1**	5' AGTTCCAGCCGTCTCTGTGT 3'	5' CTCCACGAACAGCTTCACAA 3'
**UCP-1**	5' GGGCCCTTGTAAACAACAAA 3'	5' GTCGGTCCTTCCTTGGTGTA 3'
**MYH6**	5' GAGGACCAGGCCAATGAGTA 3'	5' GCTGGGTGTAGGAGAGCTTG 3'
**MYH7**	5' TGCAGCAGTTCTTCAACCAC 3'	5' TCGAGGCTTCTGGAAGTTGT 3'
**Adiponectin**	5' GTTGCAAGCTCTCCTGTTCC 3'	5' TCTCCAGGAGTGCCATCTCT 3'
**Metallothionein-2**	5' CCATATCCCTTGAGCCAGAA 3'	5' ATCGACGAGAGATCGGTTTG 3'
**Acta-1**	5' GCATGCAGAAGGAGATCACA 3'	5' TTGTCGATTGTCGTCCTGAG 3'
**ANP**	5' CCTAAGCCCTTGTGGTGTGT 3'	5' CAGAGTGGGAGAGGCAAGAC 3'
**BNP**	5' CAGCTCTTGAAGGACCAAGG 3'	5' AGACCCAGGCAGAGTCAGAA 3'
**SERCA2a**	5' CTGTGGAGACCCTTGGTTGT 3'	5' CAGAGCACAGATGGTGGCTA 3'
**UbiC **	5' AGCCCAGTGTTACCACCAAG 3'	5' GCAAGAACTTTATTCAAAGTGCAA 3'
**GAPDH**	5' AACTTTGGCATTGTGGAAGG 3'	5' ACACATTGGGGGTAGGAACA 3'
**Rn18S **	5' CGCGGTTCTATTTTGTTGGT 3'	5' AGTCGGCATCGTTTATGGTC 3'

**Table 2.** Reagents and antibodies.

**Primary antibodies:**				
**Protein targeted**	**Host**	**Clone**	**Provider**	**Catalogue number**
SirT1	mouse	B-7	Santa Cruz Biotechnology	sc-74465
H1	goat	N-16	Santa Cruz Biotechnology	sc-34464
acetyl-H1 (Lys26)	rabbit	-	Sigma	H-7789
Adiponectin	rabbit	-	Sigma	A6354
UCP-1	rabbit		Sigma	U6382
p53	rabbit	-	Cell Signaling	#9282
acetyl-p53 (Lys382)	rabbit	-	Cell Signaling	#2525
MF-20	mouse	-	DSHB	from: Fischman, D.A.
IGF-1	goat	-	Sigma	12157
IGF-1 receptor	rabbit	-	Cell Signaling	#3027
phospho-IGF-1 receptor (Tyr1135/1136)	rabbit	-	Cell Signaling	#3024
**Secondary antibodies:**				
**Protein targeted**	**Host**		**Provider**	**Catalogue number**
HRP-conjugated anti-mouse	Goat		Amersham - GE Healthcare	NA9310V
HRP-conjugated anti-rabbit	Goat		Amersham - GE Healthcare	NA934V
HRP conjugated anti-Goat	Rabbit		Santa Cruz Biotechnology	sc-2020
Cy3-conjugated	Goat		Jackson ImmunoResearch	115-165-044
**Other reagents:**				
**Name**			**Provider**	**Catalogue number**
Sirtinol			Sigma	S7942
EX-527			Tocris Biosciences	2780
Tiron			Sigma	89460
Lipofectamin			Invitrogen	1168
ECL reagent			Amersham - GE Healthcare	RPN2209
Trizol Reagent			Invitrogen	15596
Angiotensin II			Tocris Biosciences	1158
Paraquat			Sigma	313947
SYBR®Green dye			Sigma	QR0100
mouse recombinant IGF-1			Sigma	I8879
**Plasmids:**				
**Insert**	**Plasmid**	**Source**		**References**
SirT1	pECE	Dr. Michael Greenberg-Addgene		Science 2004 Mar 26; 303 (5666):2011-2015
SirT1 H363Y	pECE	Dr. Michael Greenberg-Addgene		Science 2004 Mar 26; 303 (5666):2011-2015
Adiponectin	pGL3 basic	Dr. Bysani Chandrasekar		Mol. Cell. Biol. 2005; 25(21): 9383-9391
UCP-1 promoter	pGL3 basic	Dr. Malcolm G. Parker		J. Biol. Chem. 2008; 283: 4200-4209
Metallothionein 2a promoter	pGL3 basic	Dr. Jean-Marc Vanacker		EMBO J 1999; 15: 4270-4279
mouse mIGF-1	pIGI-1Ea	Dr. Tommaso Nastasi		PCR cloned from mouse genomic DNA into pIGI vector at restriction sites EcoRI/BamHI

## Sources of funding

This work was supported by grants of the European Union (Heart Repair: LSHM-CT-2005-018630; EUMODIC: LSHG-CT-2006-037188) and of the Foundation Leducq (Transatlantic Networks of Excellence Program: 04 CVD 03)to NR. MV is the recipient of an EIPOD (EMBL Interdisciplinary POst-Doc) fellowship.

## References

[1] Frey N, Olson EN (2003). Cardiac hypertrophy: the good, the bad, and the ugly. Annu Rev Physiol.

[2] Giordano FJ (2005). Oxygen, oxidative stress, hypoxia, and heart failure. J Clin Invest.

[3] Takimoto E, Kass DA (2007). Role of oxidative stress in cardiac hypertrophy and remodeling. Hypertension.

[4] Seddon M, Looi YH, Shah AM (2007). Oxidative stress and redox signalling in cardiac hypertrophy and heart failure. Heart.

[5] Lavu S, Boss O, Elliott PJ, Lambert PD (2008). Sirtuins--novel therapeutic targets to treat age-associated diseases. Nat Rev Drug Discov.

[6] Winn N, Paul A, Musaro A, Rosenthal N (2002). Insulin-like growth factor isoforms in skeletal muscle aging, regeneration, and disease. Cold Spring Harb Symp Quant Biol.

[7] Longo VD, Finch CE (2003). Evolutionary medicine: from dwarf model systems to healthy centenarians. Science.

[8] Andreassen M, Raymond I, Kistorp C, Hildebrandt P, Faber J, Kristensen LO (2009). IGF1 as predictor of all cause mortality and cardiovascular disease in an elderly population. Eur J Endocrinol.

[9] Reiss K, Cheng W, Ferber A, Kajstura J, Li P, Li B, Olivetti G, Homcy CJ, Baserga R, Anversa P (1996). Overexpression of insulin-like growth factor-1 in the heart is coupled with myocyte proliferation in transgenic mice. Proc Natl Acad Sci U S A.

[10] Li Q, Li B, Wang X, Leri A, Jana KP, Liu Y, Kajstura J, Baserga R, Anversa P (1997). Overexpression of insulin-like growth factor-1 in mice protects from myocyte death after infarction, attenuating ventricular dilation, wall stress, and cardiac hypertrophy. J Clin Invest.

[11] Delaughter MC, Taffet GE, Fiorotto ML, Entman ML, Schwartz RJ (1999). Local insulin-like growth factor I expression induces physiologic, then pathologic, cardiac hypertrophy in transgenic mice. Faseb J.

[12] Kajstura J, Fiordaliso F, Andreoli AM, Li B, Chimenti S, Medow MS, Limana F, Nadal-Ginard B, Leri A, Anversa P (2001). IGF-1 overexpression inhibits the development of diabetic cardiomyopathy and angiotensin II-mediated oxidative stress. Diabetes.

[13] Li Q, Yang X, Sreejayan N, Ren J (2007). Insulin-like growth factor I deficiency prolongs survival and antagonizes paraquat-induced cardiomyocyte dysfunction: role of oxidative stress. Rejuvenation Res.

[14] Hill M, Goldspink G (2003). Expression and splicing of the insulin-like growth factor gene in rodent muscle is associated with muscle satellite (stem) cell activation following local tissue damage. J Physiol.

[15] Santini MP, Tsao L, Monassier L, Theodoropoulos C, Carter J, Lara-Pezzi E, Slonimsky E, Salimova E, Delafontaine P, Song YH, Bergmann M, Freund C, Suzuki K, Rosenthal N (2007). Enhancing repair of the mammalian heart. Circ Res.

[16] Alcendor RR, Gao S, Zhai P, Zablocki D, Holle E, Yu X, Tian B, Wagner T, Vatner SF, Sadoshima J (2007). Sirt1 regulates aging and resistance to oxidative stress in the heart. Circ Res.

[17] Pillai JB, Gupta M, Rajamohan SB, Lang R, Raman J, Gupta MP (2006). Poly(ADP-ribose) polymerase-1-deficient mice are protected from angiotensin II-induced cardiac hypertrophy. Am J Physiol Heart Circ Physiol.

[18] Vahtola E, Louhelainen M, Merasto S, Martonen E, Penttinen S, Aahos I, Kyto V, Virtanen I, Mervaala E (2008). Forkhead class O transcription factor 3a activation and Sirtuin1 overexpression in the hypertrophied myocardium of the diabetic Goto-Kakizaki rat. J Hypertens.

[19] Ni YG, Wang N, Cao DJ, Sachan N, Morris DJ, Gerard RD, Kuro OM, Rothermel BA, Hill JA (2007). FoxO transcription factors activate Akt and attenuate insulin signaling in heart by inhibiting protein phosphatases. Proc Natl Acad Sci U S A.

[20] Cohen HY, Miller C, Bitterman KJ, Wall NR, Hekking B, Kessler B, Howitz KT, Gorospe M, de Cabo R, Sinclair DA (2004). Calorie restriction promotes mammalian cell survival by inducing the SIRT1 deacetylase. Science.

[21] Huffman DM, Moellering DR, Grizzle WE, Stockard CR, Johnson MS, Nagy TR (2008). Effect of exercise and calorie restriction on biomarkers of aging in mice. Am J Physiol Regul Integr Comp Physiol.

[22] Claycomb WC, Lanson NA Jr, Stallworth BS, Egeland DB, Delcarpio JB, Bahinski A, Izzo NJ Jr (1998). HL-1 cells: a cardiac muscle cell line that contracts and retains phenotypic characteristics of the adult cardiomyocyte. Proc Natl Acad Sci U S A.

[23] Calderone A, Thaik CM, Takahashi N, Chang DL, Colucci WS (1998). Nitric oxide, atrial natriuretic peptide, and cyclic GMP inhibit the growth-promoting effects of norepinephrine in cardiac myocytes and fibroblasts. J Clin Invest.

[24] Vandesompele J, De Preter K, Pattyn F, Poppe B, Van Roy N, De Paepe A, Speleman F (2002). Accurate normalization of real-time quantitative RT-PCR data by geometric averaging of multiple internal control genes. Genome Biol.

[25] Luo J, Nikolaev AY, Imai S, Chen D, Su F, Shiloh A, Guarente L, Gu W (2001). Negative control of p53 by Sir2alpha promotes cell survival under stress. Cell.

[26] Vaquero A, Scher M, Lee D, Erdjument-Bromage H, Tempst P, Reinberg D (2004). Human SirT1 interacts with histone H1 and promotes formation of facultative heterochromatin. Mol Cell.

[27] Chien KR, Knowlton KU, Zhu H, Chien S (1991). Regulation of cardiac gene expression during myocardial growth and hypertrophy: molecular studies of an adaptive physiologic response. Faseb J.

[28] Sadoshima J, Xu Y, Slayter HS, Izumo S (1993). Autocrine release of angiotensin II mediates stretch-induced hypertrophy of cardiac myocytes in vitro. Cell.

[29] Touyz R, Fareh J, Thibault G, Tolloczko B, Larivière R, Schiffrin EL (1996). Modulation of Ca2+ transients in neonatal and adult rat cardiomyocytes by angiotensin II and endothelin-1. Am J Physiol.

[30] Salas M, Vila-Petroff MG, Palomeque J, Aiello EA, Mattiazzi A (2001). Positive inotropic and negative lusitropic effect of angiotensin II: intracellular mechanisms and second messengers. J Mol Cell Cardiol.

[31] Yang H, Yang T, Baur JA, Perez E, Matsui T, Carmona JJ, Lamming DW, Souza-Pinto NC, Bohr VA, Rosenzweig A, de Cabo R, Sauve AA, Sinclair DA (2007). Nutrient-sensitive mitochondrial NAD+ levels dictate cell survival. Cell.

[32] Hingtgen SD, Tian X, Yang J, Dunlay SM, Peek AS, Wu Y, Sharma RV, Engelhardt JF, Davisson RL (2006). Nox2-containing NADPH oxidase and Akt activation play a key role in angiotensin II-induced cardiomyocyte hypertrophy. Physiol Genomics.

[33] Boudina S, Sena S, Theobald H, Sheng X, Wright JJ, Hu XX, Aziz S, Johnson JI, Bugger H, Zaha VG, Abel ED (2007). Mitochondrial energetics in the heart in obesity-related diabetes: direct evidence for increased uncoupled respiration and activation of uncoupling proteins. Diabetes.

[34] Lee Y, Gustafsson AB (2009). Role of apoptosis in cardiovascular disease. Apoptosis.

[35] Hoerter J, Gonzalez-Barroso MD, Couplan E, Mateo P, Gelly C, Cassard-Doulcier AM, Diolez P, Bouillaud F (2004). Mitochondrial uncoupling protein 1 expressed in the heart of transgenic mice protects against ischemic-reperfusion damage. Circulation.

[36] Zhou G, Li X, Hein DW, Xiang X, Marshall JP, Prabhu SD, Cai L (2008). Metallothionein suppresses angiotensin II-induced nicotinamide adenine dinucleotide phosphate oxidase activation, nitrosative stress, apoptosis, and pathological remodeling in the diabetic heart. J Am Coll Cardiol.

[37] Shibata R, Ouchi N, Ito M, Kihara S, Shiojima I, Pimentel DR, Kumada M, Sato K, Schiekofer S, Ohashi K, Funahashi T, Colucci WS, Walsh K (2004). Adiponectin-mediated modulation of hypertrophic signals in the heart. Nat Med.

[38] Pillai J, Chen M, Rajamohan SB, Samant S, Pillai VB, Gupta M, Gupta MP (2008). Activation of SIRT1, a class III histone deacetylase, contributes to fructose feeding-mediated induction of the alpha-myosin heavy chain expression. Am J Physiol Heart Circ Physiol.

[39] Miyazaki R, Ichiki T, Hashimoto T, Inanaga K, Imayama I, Sadoshima J, Sunagawa K (2008). SIRT1, a longevity gene, downregulates angiotensin II type 1 receptor expression in vascular smooth muscle cells. Arterioscler Thromb Vasc Biol.

[40] Shah B, Catt KJ (2003). A central role of EGF receptor transactivation in angiotensin II -induced cardiac hypertrophy. Trends Pharmacol Sci.

[41] Rajamohan S, Pillai VB, Gupta M, Sundaresan NR, Konstatin B, Samant S, Hottiger MO, Gupta MP (2009). SIRT1 promotes cell survival under stress by deacetylation-dependent deactivation of poly (ADP-ribose) polymerase 1. Mol Cell Biol.

[42] Vakhrusheva O, Smolka C, Gajawada P, Kostin S, Boettger T, Kubin T, Braun T, Bober E (2008). Sirt7 increases stress resistance of cardiomyocytes and prevents apoptosis and inflammatory cardiomyopathy in mice. Circ Res.

[43] Mourkioti F, Rosenthal N (2005). IGF-1, inflammation and stem cells: interactions during muscle regeneration. Trends Immunol.

[44] Qiao L, Shao J (2006). SIRT1 regulates adiponectin gene expression through Foxo1-C/enhancer-binding protein alpha transcriptional complex. J Biol Chem.

[45] Pillai J, Isbatan A, Imai S, Gupta MP (2005). Poly(ADP-ribose) polymerase-1-dependent cardiac myocyte cell death during heart failure is mediated by NAD+ depletion and reduced Sir2alpha deacetylase activity. J Biol Chem.

[46] Borradaile N, Pickering JG (2009). NAD(+), sirtuins, and cardiovascular disease. Curr Pharm Des.

